# A setback for Sustainable Development Goal 3.1: Documenting the coronavirus disease 2019 pandemic’s impact on maternal mortality through a National Confidential Enquiry in South Africa

**DOI:** 10.4102/phcfm.v18i1.5287

**Published:** 2026-04-14

**Authors:** Gomolemo Rakale, Sam T. Ntuli, Tshepo Ramarumo, Solly M. Seeletse

**Affiliations:** 1 Department of Statistical Sciences, Faculty of Science and Technology, Sefako Makgatho Health Sciences University, Pretoria, South Africa

**Keywords:** COVID-19, maternal mortality, confidential enquiry, South Africa, Sustainable Development Goals

## Abstract

**Background:**

Maternal mortality highlights health system effectiveness and social fairness. South Africa’s Confidential Enquiry into Maternal Deaths (CEMD) monitors and improves maternal healthcare. While initial decreases in maternal mortality were positive, the COVID-19 pandemic and ongoing provincial inequalities jeopardise reaching the Sustainable Development Goal (SDG) target.

**Aim:**

This analysis evaluated South Africa’s maternal mortality reduction path by examining CEMD data trends, the pandemic’s effect and provincial disparities to gauge progress towards its SDG commitments.

**Methods:**

A longitudinal trend analysis was conducted using secondary data from CEMD reports (2017–2022). The analysis focused on national and provincial institutional Maternal Mortality Ratio (iMMR) trends. Comparative analysis quantified changes and identified patterns of disparity.

**Results:**

Pre-pandemic improvement was abruptly reversed by a significant 42% surge in the national iMMR during the pandemic, underscoring the fragility of previous gains. Although a decrease was observed in 2022, the rate remained above the 2019 baseline, indicating an incomplete recovery. Furthermore, profound inter-provincial disparities were evident, with only two provinces sustaining a downward trend, the majority showing no clear improvement and three provinces consistently exhibiting exceptionally high and volatile iMMRs.

**Lessons learnt:**

South Africa is not yet on track to meet its SDG target for maternal mortality. The pandemic exposed and exacerbated systemic weaknesses, while deep-rooted provincial inequities persist. Achieving sustainable progress requires a dual strategy: building a more resilient health system capable of withstanding future shocks and implementing targeted, equity-focused interventions in underperforming regions to ensure that maternal healthcare is accessible and effective for all.

## Introduction

Maternal mortality, defined as a death during pregnancy or within 42 days of its termination,^1^ remains a critical indicator of healthcare system effectiveness and social equity. Despite global initiatives such as Millennium Development Goals (MDG),^2^ and the subsequent Sustainable Development Goals (SDG), which aim to reduce the global maternal mortality ratio (MMR) to under 70 per 100 000 live births by 2030,^3^ progress has stagnated, particularly in developing nations.^4^ South Africa (SA), despite maintaining a world-class surveillance system, the National Confidential Enquiry into Maternal Deaths (NCEMD), continues to face high MMRs,^5^ exacerbated by socioeconomic inequalities.

Established in 1999 in response to alarmingly high death rates driven initially by human immunodeficiency virus and acquired immunodeficiency syndrome (HIV/AIDS),^6,7,8^ the NCEMD has offered crucial insights into the causes, trends and preventable factors associated with maternal mortality, serving as an exemplary model for other low- and middle-income nations. Its strength lies in its near-complete capture and rigorous investigation of maternal deaths in healthcare institutions (iMMR), making its data robust for national analysis. Its primary mission is quality improvement through identifying preventable factors and disseminating evidence-based recommendations. However, COVID-19 pandemic presented an unprecedented shock that threatened to reverse prior gains^9^ and to worsen existing disparities.^10^ This study, therefore, analyses recent NCEMD data to evaluate the pandemic’s impact and to assess South Africa’s trajectory towards its SDG commitments.

## Methodology

### Study design

This study used a retrospective, longitudinal design, analysing secondary data from nationwide NCEMD reports (2017–2022). This system provides comprehensive mortality surveillance across all settings, including public and private healthcare facilities and homes.

### Study population and setting

**FIGURE 1:**
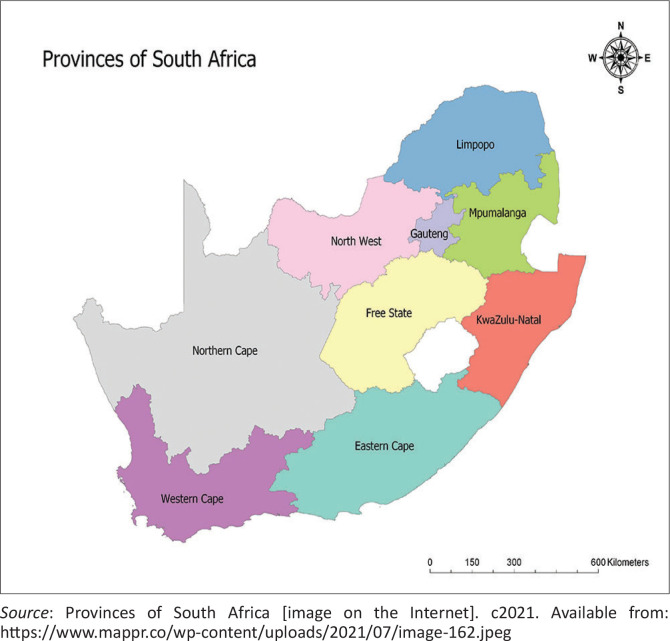
Map of South Africa.^19^

The population included all women who died while pregnant, during childbirth or within 42 days of termination of pregnancy in South Africa, as reported to the NCCEMD. The analysis focused on the public health sector across South Africa’s nine provinces (Figure 1), acknowledging the country’s two-tiered (i.e. public and private) health system.

### Data collection and analysis

Data on annual institutional Maternal Mortality Ratio (iMMR) were extracted from consolidated NCEMD reports into Microsoft Excel Spreadsheet. The key variable extracted was the institutional Maternal Mortality Ratio (iMMR). The study period was divided into three phases for comparative descriptive analysis: pre-pandemic (2017–2019) to establish a baseline, pandemic (2020–2021) to quantify disruption and post-pandemic (2022) to assess initial recovery. The national and provincial iMMR data were plotted over time (2017–2022) to visualise and describe secular trends.

### Ethical considerations

Ethical clearance to conduct this study was obtained from the Sefako Makgatho University Research Ethics Committee. The ethical clearance number is SMUREC/S/481/2025:PG and authorised the use of the archival data without individual consent. All analyses adhered to strict confidentiality, utilising only aggregated data with no personal identifiers.

## Results

### National trends

The national data revealed a distinct J-curve pattern in maternal mortality. The rate declined consistently before the pandemic, reaching a low in 2019. This progress was abruptly reversed during the COVID-19 pandemic, with the rate peaking sharply in 2021, a 49.9% increase from the 2019 baseline. Although a significant recovery followed in 2022, the rate remained elevated above the pre-pandemic low, indicating an incomplete rebound (Table 1).

**TABLE 1:** Detailed National Analysis of the Maternal Mortality Data.

Year	Number of deaths	iMMR (per 100 000 live births)	Interpretation
2017	1222	134.9	Baseline period, showing a high maternal mortality burden.
2018	1169	122.9	A slight improvement, suggesting positive efforts in maternal healthcare.
2019	1056	98.8	A significant low point. This represents the best performance in the period, potentially reflecting the success of focused maternal health programmes.
2020	1183	126.1	A sharp reversal of the previous year’s gains. This aligns with the start of the COVID-19 pandemic, which disrupted healthcare access and diverted resources.
2021	1497	148.1	The peak of the crisis. The full impact of the pandemic is seen, with a 49.9% increase in the iMMR from 2019. The COVID-19 is a known risk factor for severe pregnancy outcomes.
2022	1062	109.9	A dramatic improvement indicates a strong recovery as pandemic pressures eased. However, the rate is still higher than the 2019 rate, suggesting ongoing challenges.

*Source*: Adapted from Moodley J, Pattinson RC, Fawcus S, et al. The confidential enquiry into maternal deaths in South Africa: A case study. BJOG. 2014;121(Suppl 4):53–60. https://doi.org/10.1111/1471-0528.12869

COVID-19, coronavirus pandemic 2019; iMMR, institutional Maternal Mortality Rate.

### Provincial trends

**FIGURE 2:**
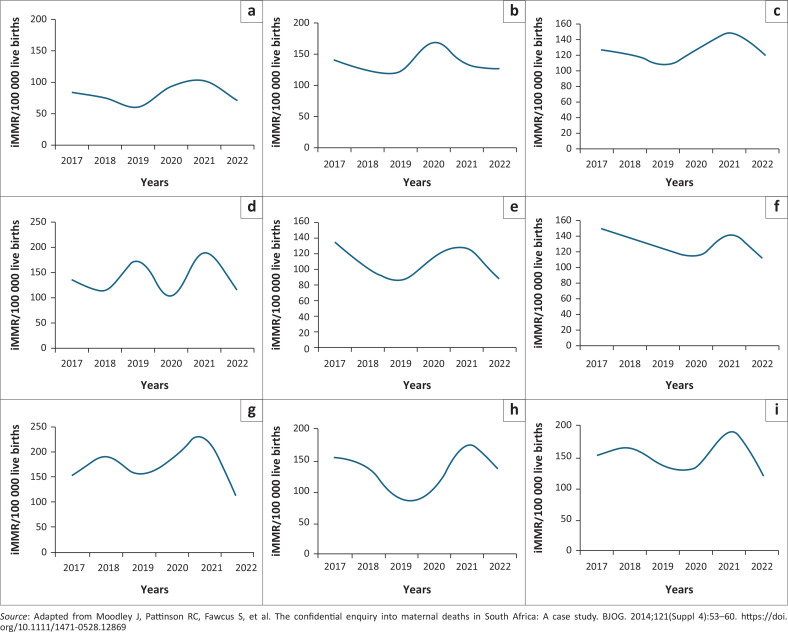
Provincial Maternal Mortality Data for the period 2017-2022 in the respective provinces of South Africa: (a) Western Cape; (b) Eastern Cape; (c) Gauteng; (d) Northern Cape; (e) KwaZulu-Natal; (f) Limpopo; (g) Free State; (h) Mpumalanga; (i) North West.

Analysis of South Africa’s nine provinces revealed starkly different maternal mortality trends, leading to their classification into three distinct groups based on their iMMR (2017–2022): consistent improvements; persistently high and unstable and mixed or stagnant performers (Figure 2).

#### Consistent improvements

Two provinces exhibited a distinct downward trajectory or consistently maintained lower-than-average rates. KwaZulu-Natal demonstrated sustained and significant improvement, reducing its iMMR from 135.7 in 2017 to 87.8 in 2022. Limpopo showed a clear, if less dramatic, improving trend from 150.2 in 2017 to 114.7 in 2022.

#### Persistently high and unstable

These provinces present a serious and ongoing challenge to South Africa’s maternal healthcare system. The Free State consistently has the highest and most volatile iMMR in the country. A decrease in 2022 is viewed with caution because of the province’s history of instability and a very vulnerable system. After years of high rates, Northwest saw a substantial increase in 2021. Despite some recovery in 2022, it remains a major concern. Mpumalanga experienced a significant peak in 2021 and continues to have one of the country’s highest iMMRs, indicating persistent systemic issues.

#### Mixed or stagnant performers

The analysis reveals stark provincial disparities in maternal mortality trends. The Eastern Cape and Gauteng showed little to no sustained improvement, with both remaining significantly impacted post-pandemic. In contrast, the Western Cape demonstrated notable resilience, recovering quickly to achieve the country’s lowest rate by 2022, underscoring the strength of its healthcare system.

Finally, the Northern Cape presents a unique case of extreme volatility. Its data are erratic, and it recorded the nation’s highest iMMR in 2021, likely because of the immense difficulty of providing consistent healthcare across its vast, sparsely populated geography.

## Discussion

Our analysis identified three distinct phases in South Africa’s maternal mortality trends. The iMMR during the pre-pandemic period showed a clear improvement, suggesting that targeted interventions and programming, such as ESMOE and BANC, were indeed having a positive and measurable impact,^11,12^ though the role of enhanced data reporting cannot be discounted. While these advancements are promising, some argue that the improvements might not be sustainable, especially given the ongoing challenges within the healthcare system.^13^

The COVID-19 pandemic significantly reversed previous progress, resulting in a 49.9% rise in the national iMMR. This setback is consistent with global trends observed in countries such as Uganda,^14^ Kazakhstan,^15^ and Kenya.^16^ The alarming surge, however, might be a temporary deviation attributable to the extraordinary pressure on healthcare systems during the early stages of the pandemic, rather than an indicator of a fundamental and enduring decline in maternal healthcare. In the post-pandemic period (2022), the iMMR decreased but remained above the pre-pandemic baseline of 98.8 in 2019. Similarly, a study conducted in Kenya indicated a decrease in the national iMMR to 99 per 100 000 live births during the same year.^16^ Our findings suggest an incomplete recovery and indicate the pandemic may have exacerbated underlying weaknesses in the healthcare system.

Finally, the analysis revealed profound and alarming provincial disparities, findings that are consistent with a previous study conducted in Kenya.^16,17^ Our study indicated that two provinces demonstrated a clear downward trend or maintained lower-than-average rates. While factors such as improved quality of emergency obstetric care, better staffing, and more reliable supplies of essential medicines are potential drivers for the decline in these two provinces, the observed trend may also reflect advancements in data collection and reporting mechanisms.^13,18^ In contrast, three provinces exhibited consistently high and volatile iMMRs, pointing to critical public health challenges potentially influenced by socioeconomic factors and systemic instability, while the majority showed no clear progress.

### Limitations

The analysis acknowledged its ecological nature, describing population-level associations rather than establishing individual causality. Furthermore, the observed trends could be influenced by concurrent improvements in data collection and reporting completeness over time, not solely by changes in the actual number of deaths.

## Conclusion

This study concludes that the COVID-19 pandemic significantly reversed South Africa’s maternal health progress and exposed critical systemic weaknesses. With an incomplete recovery and stark provincial inequalities, the country is off-track to meet its maternal mortality reduction goal (SDG 3.1). Recovery depends on a dual approach: strengthening the health system’s overall resilience and implementing targeted, equitable interventions in struggling provinces. Building a robust system requires learning from both pre-pandemic successes and the hard lessons of the pandemic.
